# The effect of cigarette smoking use and cessation on serum insulin-like growth factors

**DOI:** 10.1038/sj.bjc.6602150

**Published:** 2004-08-31

**Authors:** A G Renehan, W S Atkin, S T O'Dwyer, S M Shalet

**Affiliations:** 1 Department of Surgery, Christie Hospital NHS Trust, Wilmslow Road, Manchester M20 4BX, UK; 2 Cancer Research UK Colorectal Unit, St Mark's Hospital, London, UK; 3 Department of Endocrinology, Christie Hospital NHS Trust, Manchester, UK

**Keywords:** insulin-like growth factors, IGF-binding proteins, cancer risk, cigarette smoking

## Abstract

The patterns of risk association between circulating levels of insulin-like growth factor (IGF)-I, and its main binding protein, IGFBP-3, differ between smoking and nonsmoking-related cancers. To investigate this observation further, we measured serum IGF-I, IGF-II and IGF-binding protein-3 concentrations in 232 men and 210 women (aged 55–64 years), and related peptide levels to smoking characteristics. Current smoking was associated with significant reductions in mean IGFBP-3 levels in men assessed by the number of cigarettes smoked daily (*P*_trend_=0.007) and pack-years smoked (*P*_trend_=0.03). Mean IGF-I levels decreased with increasing cigarette use in men (*P*_trend_=0.11). There were no patterns of association between smoking and IGF peptides in women. For male former *vs* never smokers, there were no differences in mean IGF-I and IGFBP-3 concentrations, suggesting that smoking cessation is associated with normalisation of peptide concentrations.

Insulin-like growth factor-one (IGF-I) is a multifunctional regulatory peptide important in tumour cell growth and survival (Jones and Clemmons, 1995). In the circulation, IGF-I is predominantly bound (>90%) to the major insulin-like growth factor-binding protein, IGFBP-3 (Clemmons, 1997). Unlike most other growth factors, the IGFs have classical endocrine as well as local paracrine influences on cell behaviour (Rajaram *et al*, 1997). Concentrations of circulating total IGF-I and IGFBP-3 are influenced by growth hormone, age (levels decline with age after puberty), gender and nutritional status (Thissen *et al*, 1994; Juul, 2003). Nevertheless, measurement of circulating IGF peptides levels can be used as a marker of the general body stores (Holly and Hughes, 1994).

Across the general population, there are wide interindividual variations in IGF-I and IGFBP-3 concentrations, which may impact upon cancer risk (Pollak, 2000; Yu and Rohan, 2000). In a recent systematic review and meta-regression analysis of 21 studies, we determined the associations between circulating IGF-I and IGFBP-3 levels and cancer risk (Renehan *et al*, 2004), and demonstrated that the patterns of association differed between smoking and nonsmoking-related cancer. Specifically, total IGF-I concentrations are positively associated with the risk of prostate, colorectal and pre-menopausal breast cancers, but not lung cancer, while total IGFBP-3 concentrations are positively associated with the risk of pre-menopausal breast cancer, and, after excluding a recruitment-bias study, inversely associated with lung cancer risk. A further population-based study reported that IGFBP-3 concentrations are inversely associated with increased risk of lung cancer mortality, but noted no association with serum IGF-I levels (Wakai *et al*, 2002). In light of these epidemiological observations, we hypothesised that cigarette smoking may influence IGF physiology. Thus, the aim of this study was to determine the relationships of serum IGF-I, IGF-II and IGFBP-3 with characteristics of smoking exposure.

## MATERIALS AND METHODS

### Study design

Using a cross-sectional design, we studied 232 men and 210 women attending one centre (1998–99) within the Flexi-Scope colorectal cancer screening trial (Flexi-Scope-Trial-Collaborators, 2002). Participants were healthy ambulatory individuals aged 55–64 years, invited by open invitation from general medical practitioner registries. With Ethics Committee approval and after obtaining informed consent, a trained researcher interviewed participants. Smoking exposure was evaluated using a modification of the European Prospective Investigation into Cancer (EPIC) study questionnaire (Sargeant *et al*, 2001), and individuals categorised as never, former and current smokers. Computed exposure variables included pack-years smoked, that is, number of packs (one pack=20 cigarettes) smoked per day multiplied by the number of years smoked. Participants were questioned about medical history and defined as having major illness in accordance with EPIC study criteria (Sargeant *et al*, 2001) (see footnote to Table 1

**Table 1:** Characteristics of 232 men and 210 women, aged 55–64 years

	**Never smokers**	**Former smokers**	**Current smokers**
*Men*			
Number of participants	91	96	45
Mean (s.d.)			
Age (years)	60.8 (2.8)	60.8 (2.8)	60.5 (2.6)
Height (cm)	175 (7.1)	176.1 (6.5)	175 (6.8)
Weight (kg)	80.4 (13.6)	82.6 (10.6)	79.6 (12.4)
BMI (kg m^−2^)^a^	26.3 (4.5)	26.6 (3.2)	26.0 (3.4)
Age started smoking (years)	—	17.2 (3.3)	20.1 (7.5)
Duration smoking (years)	—	22.5 (9.7)	40.4 (8.1)
Cigarettes per day	—	20.4 (14.4)	17.0 (10.0)
Pack-years of cigarette smoking^b^	—	23.7 (20.2)	33.9 (20.5)
Age when quit smoking (years)	—	39.6 (9.2)	—
Numbers (%)			
Caucasian	86 (95)	87 (91)	41 (91)
Current aspirin use	15 (17)	18 (19)	9 (20)
Major illness^c^	23 (25)	42 (44)	12 (27)
Diabetes mellitus	3 (3)	9 (9)	1 (2)
			
*Women*			
Number of participants	110	60	40
Mean (s.d.)			
Age (years)	59.7 (2.7)	60.5 (2.7)	59.6 (2.6)
Height (cm)	162 (6)	162 (7)	163 (7.1)
Weight (kg)	69.5 (10.8)	70.8 (13.9)	68.2 (10.1)
BMI (kg m^−2^)^a^	26.6 (4.3)	26.9 (5.3)	25.8 (3.9)
Age started smoking (years)	—	19.5 (3.9)	19.9 (8.3)
Duration smoking (years)	—	21.0 (10.4)	39.5 (8.5)
Cigarettes per day	—	12.4 (6.7)	13.9 (7.9)
Pack-years of cigarette smoking^b^	—	13.9 (12.5)	28.3 (18.2)
Age when quit smoking (years)	—	40.5 (10.3)	—
Numbers (%)			
Caucasian	106 (96)	58 (97)	40 (100)
Current aspirin use	13 (12)	5 (8)	5 (13)
Major illness^c^	29 (26)	14 (23)	9 (23)
Diabetes mellitus	3 (3)	2 (2)	1 (3)
Current HRT user^d^	42 (38)	23 (38)	18 (45)
Ever HRT user	50 (46)	29 (48)	20 (50)

s.d.=standard deviation.

a BMI=body mass index.

b Pack-years presented as median (inter-quartile range).

c Major illness included: high blood pressure (hypertension) requiring treatment with drugs, high blood cholesterol (hyperlipidemia), angina, heart attack (myocardial infarction), stroke, other vascular disease (peripheral vascular disease), diabetes mellitus (excluding gestational diabetes) and cancer.

d HRT=hormonal replacement therapy; Current use defined as within the past 6 months.

). Hormonal replacement therapy (HRT) use in women was also recorded. Details of alcohol consumption, physical activity and diet were not available. For each participant, height and weight were measured, and body mass index (BMI) accordingly calculated as weight/height^2^ (kg m^−2^).

### Blood collection

Blood was obtained in clotted tubes and immediately transported to the laboratory. Serum was isolated by centrifugation at 3000 r.p.m. for 10 min at room temperature and stored at −80°C before analyte determination. Within the study, several quality control tests were performed, which demonstrated that: (i) repeated analyte sampling over short periods in healthy individuals showed minimal variation; (ii) time from venepuncture to processing had little impact and (iii) there was long-term stability at −80°C storage (Renehan, 2004).

### Measurements of IGF-I, IGF-II and IGFBP-3

Serum IGF-I concentrations were measured, following acid–alcohol extraction, by an established in-house radioimmunoassay (Renehan *et al*, 2000b, 2001). Serum IGF-II and IGFBP-3 levels were determined using a commercially available immuno-radiometric assays kit (Diagnostic Systems Laboratories, Inc. Webster, TX, USA). All determinants were measured in duplicate blind to cigarette and gender status. The IGF-I/IGFBP-3 molar ratio was calculated using the conversion: 1 ng ml^−1^ is 0.130 nmol l^−1^ for IGF-I and 0.036 nmol l^−1^ for IGFBP-3. The coefficients of variation (CVs) for intra- and inter-assay testing were less than 5 and 10%, respectively (Renehan *et al*, 2003).

### External validity

Studies from the Flexi-Scope Trial have shown that the distribution across social classes is broadly representative of the general population (McCaffery *et al*, 2002). In addition, baseline characteristics of this study cohort by smoking status were similar to those reported for age-matched UK populations (Appendices A1 and B1).

### Statistical analysis

Data were analysed separately for men and women as we previously reported significant differences in mean IGF-I, IGF-II and IGFBP-3 concentrations by gender (Renehan *et al*, 2000b). All analytes were parametrically distributed (Kolmogorov–Smirnov test), and thus the principal results were expressed as means and standard deviations (s.d.). For descriptive analysis, Student's *t*-tests, one-way ANOVA and chi-squared (*χ*^2^) tests were used.

With smoking characteristics as the principal factor of interest, we evaluated for trends across serum IGF concentrations using linear regression models. As factors of interest may have trends in opposite directions in current *vs* former smokers (e.g. BMI) (Chao *et al*, 2000; DoH, 2000; Sargeant *et al*, 2001), we analysed the data separately for never (referent) *vs* current smokers, and never *vs* former smokers. As the distributions for quantifying smoking exposure were not continuous – for example, participants tended to report the number of cigarettes smoked per day in multiples of five – we determined the ranks for these variables based on arbitrary cutoff points. Thus, for instance, the average number of cigarettes per day was ranked as 1, 2 and 3, for <5, 15–24 and ⩾25 cigarettes smoked per day, respectively. Never smokers were then denoted as zero and models constructed. Model A (univariate) was unadjusted with dependent variables IGF-I, IGF-II, IGFBP-3 and the IGF-I/IGFBP-3 molar ratio. Model B was adjusted for age and ethnicity as IGF levels decline with age after puberty (Juul, 2003) and vary between ethnic groups (Platz *et al*, 1999). We included both BMI and height in this model to capture information on both body composition and body size. For women, we included current (within past 6 months) use of HRT as its use is associated with reductions in mean IGF-I and IGFBP-3 concentrations (Leung *et al*, 2004). To accommodate the opposing effects of IGF-I and IGFBP-3 (*r*=0.59, *P*<0.001), and IGF-II and IGFBP-3 (*r*=0.61, *P*<0.001), model C included adjustments for IGFBP-3 where IGF-I and IGF-II were dependent variables, and for IGF-I where IGFBP-3 was the dependent variable. Results were reported as *β* coefficients, and their standard errors (s.e.) and the total model *r*^2^ were calculated to provide a sense of the model variability and strength of fit (STATA version 7.0: StataCorp, College Station, TX, USA).

## RESULTS

The study baseline characteristics are shown in Table 1. Of the 442 participants, 19% of men and 19% of women were current smokers; 41% of men and 29% of women were former smokers at the time of blood sampling. As reported in other studies (Chao *et al*, 2000; DoH, 2000; Sargeant *et al*, 2001), current smoking was associated with lower BMI values in both genders, while former smoking was associated with higher BMI values, compared to that for never smoking. Men tended to start smoking at an earlier age, smoke more cigarettes per day and had greater pack-years of smoking, compared with women. In all, 40% (83 out of 210) of women were current HRT users.

The mean concentrations for serum IGF-I, IGF-II, IGFBP-3 and calculated IGF-I/IGFBP-3 molar ratio according to gender and smoking status are shown in Table 2

**Table 2:** Serum concentrations of IGF-I, IGF-II, IGFBP-3 and the molar IGF-I/ IGFBP-3 ratio by smoking status in men and women

	**Mean concentrations (s.d.)**					
	**IGF-I (ng ml^−1^)**		**IGF-II (ng ml^−1^)**	**IGFBP-3 (ng ml^−1^)**		**IGF-I/IGFBP-3**
*Men*						
Smoking status						
Never smoker	200 (62)		833 (181)	3192 (549)		0.225 (0.053)
Former smoker	189 (73)		834 (205)	3099 (657)		0.219 (0.059)
Current smoker	183 (67)		806 (192)	2903 (615)		0.229 (0.061)
*P* (1-ANOVA)	0.32		0.71	0.04		0.57
				
*Women*						
HRT within 6 months						
No (*n*=127)	178 (52)		883 (187)	3355 (588)		0.198 (0.050)
Yes (*n*=83)	156 (50)		877 (189)	3134 (636)		0.186 (0.048)
*P* (Student's *t*-test)	0.003		0.81	0.01		0.09
Smoking status	No HRT	HRT		No HRT	HRT	
Never smoker	181 (56)	163 (54)	892 (194)	3368 (611)	3142 (603)	0.198 (0.051)
Former smoker	176 (52)	149 (37)	868 (174)	3388 (611)	3145 (607)	0.188 (0.046)
Current smoker	172 (44)	151 (55)	868 (190)	3258 (483)	3101 (774)	0.191 (0.50)
*P* (1-ANOVA)	0.77	0.51	0.65	0.69	0.97	0.43

s.d.=standard deviation. HRT=hormonal replacement therapy. Current HRT use defined as within the past 6 months.

Molar ratio conversion: 1 ng ml^−1^ is 0.130 nmol l^−1^ for IGF-I and 0.036 nmol l^−1^ for IGFBP-3.

. With never smokers as referents, mean levels for IGF-I were higher (mean difference=26.1, 95% confidence interval, 9.7–42.4 ng ml^−1^), IGF-II were lower (−59.3, −112.0 to −6.7 ng ml^−1^), IGFBP-3 were lower (−89.6, −253.3 to 74.1 ng ml^−1^), and IGF-I/IGFBP-3 ratio were higher (0.028, 0.007–0.013) in men compared with women. Among men, smoking was associated with nonsignificant reductions in mean serum IGF-I levels, but significant reductions in mean IGFBP-3 levels (1-ANOVA, *P*=0.04). As expected, the current use of HRT in women was associated with significant reductions in mean serum IGF-I (Student's *t*-test, *P*=0.003) and IGFBP-3 (*P*=0.01) concentrations. After taking account of HRT status, there was no significant association between mean IGF-I or IGFBP-3 concentrations and smoking habit in women. There were no distinct patterns of association between smoking and serum IGF-II.

We evaluated for trends in IGF peptide concentrations and smoking exposure (Table 3

**Table 3:** Serum concentrations of IGF-I, IGF-II, IGFBP-3 and the molar IGF-I/ IGFBP-3 ratio by smoking characteristics in men and women

		**Mean concentrations (s.d.)**			
	***n***	**IGF-I (ng ml^−1^)**	**IGF-II (ng ml^−1^)**	**IGFBP-3 (ng ml^−1^)**	**IGF-I/IGFBP-3**
*Men*					
Number of cigarettes daily					
Never (zero)	91	200 (62)	833 (181)	3192 (549)	0.225 (0.053)
1–14 cigarettes	18	180 (68)	799 (209)	2959 (562)	0.220 (0.065)
15–24 cigarettes	17	190 (54)	799 (165)	2921 (714)	0.237 (0.053)
⩾25 cigarettes	10	178 (77)	831 (211)	2771 (566)	0.233 (0.071)
Model A, *P* for trend		0.20	0.61	0.005	0.51
Model B, *P* for trend		0.11	0.43	0.002	0.62
Model C, *P* for trend		0.76	0.14	0.007	—
Current smokers, pack-years					
Never (zero)	91	200 (62)	833 (181)	3192 (549)	0.225 (0.053)
1–20	15	181 (70)	839 (179)	2929 (623)	0.223 (0.064)
21–39	13	184 (67)	690 (194)	2794 (596)	0.237 (0.063)
⩾40	17	185 (61)	867 (172)	2963 (649)	0.229 (0.061)
Model A, *P* for trend		0.22	0.65	0.02	0.66
Model B, *P* for trend		0.15	0.48	0.009	0.70
Model C, *P* for trend		0.87	0.24	0.03	—
					
*Women*					
Number of cigarettes daily					
Never (zero)	110	174 (56)	892 (194)	3282 (615)	0.192 (0.050)
1–9 cigarettes	10	154 (47)	883 (124)	3347 (403)	0.168 (0.039)
10–19 cigarettes	17	165 (49)	873 (191)	3221 (576)	0.194 (0.050)
⩾20 cigarettes	13	167 (56)	850 (237)	3020 (811)	0.209 (0.054)
Model A, *P* for trend		0.42	0.43	0.21	0.95
Model B, *P* for trend		0.49	0.46	0.18	0.79
Model C, *P* for trend		0.94	0.86	0.25	—
Current smokers, pack-years					
Never (zero)	110	174 (56)	892 (194)	3282 (615)	0.192 (0.050)
1–18	13	163 (46)	893 (137)	3345 (473)	0.175 (0.043)
19–38	14	159 (51)	857 (194)	3196 (558)	0.179 (0.048)
⩾39	13	167 (56)	850 (237)	3020 (811)	0.203 (0.052)
Model A, *P* for trend		0.35	0.38	0.19	0.95
Model B, *P* for trend		0.47	0.41	0.17	0.81
Model C, *P* for trend		0.94	0.92	0.24	—

s.d.=standard deviation.

Pack-years were divided into tertiles based on the distribution of all current smokers for men and women.

Multiple linear regression model A: unadjusted.

Model B: adjusted for age, ethnicity, height, body mass index and current HRT status in women.

Model C: adjusted for age, ethnicity, height, body mass index, current HRT status in women and IGFBP-3 for IGF-I and IGF-II as dependent variables, and IGF-I for IGFBP-3 as dependent variable.

). Among male current smokers, and taking never smokers as zero cigarettes, there were significant reductions in mean IGFBP-3 concentrations assessed as the number of cigarettes smoked per day (unadjusted: *β*=−144, s.e.=51, *P*_trend_=0.005, *r*^2^=0.057) and as pack-years smoked (*β*=−108, s.e.=46, *P*_trend_=0.02, *r*^2^=0.040). These significant trends remained after adjustments for age, ethnicity, height, BMI and IGF-I (fully adjusted: *β*=−113, s.e.=41, *P*_trend_=0.007, *r*^2^=0.421 and *β*=−82, s.e.=37, *P*_trend_=0.03, *r*^2^=0.410, respectively). Among female current smokers, there were nonsignificant reductions in mean IGFBP-3 levels with increasing smoking exposure (fully adjusted: *β*=−48, s.e.=42, *P*_trend_=0.25, *r*^2^=0.356 and *β*=−51, s.e.=43, *P*_trend_=0.24, *r*^2^=0.356 for cigarettes per day and pack-years, respectively). For men, there was a nonsignificant trend towards reduced mean IGF-I levels with increasing smoking exposure (*P*_trend_=0.11), but no association after adjustment for IGFBP-3. There were no trends for IGF-I in women, and IGF-II or the IGF-I/IGFBP-3 ratio in both genders.

Data from the UK Doctor's study (Peto *et al*, 2000) have shown that the risk of smoking-related cancers returns towards general population risk levels with increasing duration since smoking cessation. To test the relevance of this observation to circulating IGFs, we evaluated the trends in mean analyte levels by categories of years since quit smoking, and age when quit smoking (Table 4

**Table 4:** Serum concentrations of IGF-I, IGF-II, IGFBP-3 and the molar IGF-I/ IGFBP-3 ratio by past smoking characteristics (in ex-smokers) in men and women

		**Mean concentrations (s.d.)**			
	***n***	**IGF-I (ng ml^−1^)**	**IGF-II (ng ml^−1^)**	**IGFBP-3 (ng ml^−1^)**	**IGF-I/IGFBP-3**
*Men*					
Years since quit smoking					
Never (zero)	91	200 (62)	833 (181)	3192 (549)	0.225 (0.053)
⩾20 years	56	183 (55)	813 (196)	3098 (655)	0.214 (0.050)
10–19 years	26	207 (93)	843 (174)	3128 (638)	0.236 (0.072)
Less than 10 years	14	179 (95)	899 (280)	3044 (742)	0.209 (0.069)
*P* for trend^a^		0.71	0.17	0.40	0.58
Age when quit smoking					
Never	91	200 (62)	833 (181)	3192 (549)	0.225 (0.053)
Before aged 35 years	36	188 (59)	794 (213)	3168 (722)	0.215 (0.052)
Aged 35–44 years	31	198 (88)	876 (156)	3150 (567)	0.226 (0.072)
Aged 45 years and after	29	179 (72)	838 (235)	2958 (665)	0.217 (0.055)
*P* for trend^a^		0.67	0.02	0.39	0.85
					
*Women*					
Current smokers, pack-years					
Never (zero)	110	174 (56)	892 (194)	3282 (615)	0.198 (0.051)
⩾20 years	33	164 (51)	861 (184)	3269 (651)	0.188 (0.053)
10–19 years	13	176 (49)	868 (173)	3461 (600)	0.188 (0.034)
Less than 10 years	14	160 (46)	884 (161)	3201 (553)	0.187 (0.044)
*P* for trend^b^		0.24	0.49	0.62	0.21
Age when quit smoking					
Never	110	174 (56)	892 (194)	3282 (615)	0.198 (0.051)
Before age 35 years	22	164 (51)	881 (190)	3334 (700)	0.185 (0.048)
Aged 35–44 years	18	166 (55)	827 (183)	3261 (653)	0.189 (0.053)
Aged 45 years and after	20	167 (42)	891 (148)	3283 (502)	0.190 (0.040)
*P* for trend^b^		0.31	0.39	0.70	0.28

s.d.=standard deviation.

a Models adjusted for age, ethnicity, height, body mass index and IGFBP-3 for IGF-I and IGF-II as dependent variables, and IGF-I for IGFBP-3 as dependent variable.

b Models adjusted for age, ethnicity, height, body mass index, current HRT status and IGFBP-3 for IGF-I and IGF-II as dependent variables, and IGF-I for IGFBP-3 as dependent variable.

). In general, mean values for serum IGF-I, IGF-II, IGFBP-3 and IGF-I/IGFBP-3 ratios demonstrated no difference among former smokers compared to current smokers. Mean IGFBP-3 levels in men who recently stopped smoking were lower than those in never smokers, but this did not reach statistical significance.

## DISCUSSION

Cigarette smoking was associated with significant exposure-related reductions (up to 13%) in mean serum IGFBP-3 levels in men (and to a lesser extent in women), an observation which may be relevant for smoking-related tumour development. Smoking tended to decrease mean IGF-I levels in men, but these changes may reflect parallel reductions in IGFBP-3 concentrations. Mean IGF-I and IGFBP-3 levels were similar for former smokers and never smokers, suggesting that these markers of cancer risk normalise following smoking cessation.

An advantage of this study was the narrow age-defined population-based cohort, as we and others have shown that circulating levels of IGF peptides change over a wide age range in a nonlinear fashion (Renehan *et al*, 2000a; Juul, 2003). Comparing never *vs* current smokers, and never *vs* former smokers, was another key study feature, as factors of interest may influence IGF physiology in opposite directions in different smoking groups. In addition, in previous studies, smoking exposure has often been treated as an ordinal scale variable (never, former smoker, current smoker) without taking account of quantities smoked and the time period of exposure. Having taken account of these factors, the current study demonstrated significant exposure-related trends in IGFBP-3 levels.

The relatively small numbers in the smoking categories and the cross-sectional design with once-only analyte measurements were potential disadvantages of the study. Further limitations were the lack of data for alcohol consumption, dietary factors and physical activity. For alcohol consumption, a factor known to be associated with cigarette smoking, studies have shown inconsistent relationships with serum IGF-I concentrations – increase (Goodman Gruen and Barrett Connor, 1997; Kaklamani *et al*, 1999), decrease (Teramukai *et al*, 2002) or no change (Holmes *et al*, 2002a) – but mainly positive correlations with IGFBP-3 (Holmes *et al*, 2002a; Teramukai *et al*, 2002). Recent reports suggest that plant-based diets (Holmes *et al*, 2002b), high-protein diets (Allen *et al*, 2003) and milk consumption (Ma *et al*, 2001; Gunnell *et al*, 2003) may be important determinants of circulating IGF peptide levels. However, cross-sectional studies fail to demonstrate consistent associations between circulating IGF peptides and level of physical activity (Landin Wilhelmsen *et al*, 1994; Voskuil *et al*, 2001; Holmes *et al*, 2002a; Teramukai *et al*, 2002). These need to be considered in future studies.

One other study (Kaklamani *et al*, 1999) has specifically determined the relationships between serum IGF peptides and smoking, but was limited to 130 individuals (only 22 current smokers) across a wide age range, men and women analysed together, and the regression analyses of relationships with smoking exposure (as a continuous variable) were limited to current smokers only, without taking account of never smokers (i.e. zero). Additional studies have determined IGF–smoking relationships within wider analyses of associations with lifestyle and/or anthropometric factors. Despite these differences in design, there are emerging consistent observations: (i) across different populations – Japanese (Teramukai *et al*, 2002), Greek (Kaklamani *et al*, 1999), American (Chang *et al*, 2002), United Kingdom (present study) – there are reductions in mean IGFBP-3 levels with smoking in men, but not in women (Chang *et al*, 2002; Holmes *et al*, 2002a); (ii) associations between serum IGF-I and cigarette smoking are generally inverse in men (Landin Wilhelmsen *et al*, 1994; Teramukai *et al*, 2002), but weak (Holmes *et al*, 2002a) or absent (Landin Wilhelmsen *et al*, 1994) in women. However, in post-menopausal women, the expected reductions in mean serum IGF-I levels associated with HRT usage may be greater among current smokers (Chang *et al*, 2002). Unique to our study, we showed that the trend towards reduced mean IGF-I levels with increasing smoking was attenuated after adjustment for IGFBP-3, suggesting that these changes may be dependent on parallel reductions in IGFBP-3 concentrations.

What are the implications for cancer mechanisms? For lung cancer, the findings of the current study are consistent with our meta-analysis (Renehan *et al*, 2004) that reported no association with circulating IGF-I but a significant inverse association with IGFBP-3 (after excluding a heavy smokers-only study). An inverse role for IGFBP-3 in lung tumorigenesis is supported by the observation that constitutive expression of IGFBP-3 inhibits the growth of non-small-cell lung cancer (Lee *et al*, 2002). Yet, for pre-menopausal breast cancer, there is a positive association between circulating IGFBP-3 and cancer risk (Renehan *et al*, 2004). These apparent paradoxes are not unexpected as the cellular functions of IGFBP-3 are multi-directional and, depending on the cellular environment, may be inhibitory (through sequestration of IGF ligand), antiproliferative and proapoptotic (Firth and Baxter, 2002) or antiapoptotic (McCaig *et al*, 2002) via IGF-independent pathways. Whereas some authors (Pollak, 2000; Yu and Rohan, 2000) have hypothesised that the relative levels of IGF-I to IGFBP-3 may be important for cancer risk, the *absolute* quantities (reflecting total body stores) may be more pertinent, remembering that IGFBP-3 circulates in molar concentrations considerably (five-fold) greater than IGF-I.

The study findings suggest (albeit indirectly) that serum IGF-I and IGFBP-3 levels normalise after smoking cessation, an observation that is clearly relevant to cancer prevention. However, a clear understanding of the ‘ups and downs’ of IGF-I and IGFBP-3 is required. Thus, for example, cancer prevention trials (McTiernan, 2003) are currently being designed to modulate circulating IGF-I and IGFBP-3 as biomarkers of cancer risk, and paradoxical results may be predicted for nonsmokers *vs* smokers – an increase in serum IGF-I levels after smoking cessation may simply reflect peptide normalisation rather than represent a prediction of increased cancer risk.

The reasons why smoking induces reductions in IGFBP-3 levels and why there are gender differences are unclear. However, the merit of this study is that it focuses attention on smoking as a modifiable influence of circulating IGF peptides, surrogate markers of common cancer risk.

## Appendix A1

A comparison of the baseline characteristics of the study participants with UK population is given in Table A1

**Table A1:**

	**Manchester study (55–64 years)**	**UK population (55–64 years) (Health Survey for England)**	***P***-**value**^a^
*Numbers* (%)			
Men	*N*=232	*N*=985^b^	
Never smoker	91 (39)	315 (32)	0.04
Former smokers	96 (41)	443 (45)	0.36
Current smokers	45 (19)	226 (23)	0.24
			
Women	*N*=210	*N*=1147^b^	
Never smoker	110 (52)	573 (50)	0.57
Former smokers	60 (29)	287 (25)	0.32
Current smokers	40 (19)	287 (25)	0.08
			
*Number of cigarettes smoked in current smokers*			
Men	*N*=232	*N*=231^c^	
Under 10 cigarettes	32 (14)	42 (18)	
10 to under 20 cigarettes	84 (36)	85 (37)	
20 and over	116 (50)	104 (45)	
Mean (s.e.)	17.0 (1.5)	18.2 (0.75)	
			
Women	*N*=210	*N*=289^c^	
Under 10 cigarettes	67 (27)	72 (25)	
10 to under 20 cigarettes	80 (38)	124 (43)	
20 and over	73 (35)	93 (32)	
Mean (s.e.)	13.9 (1.4)	14.3 (0.50)	

Values in parentheses are percentages unless otherwise stated.

s.e.=standard error.

a *χ*^2^ test.

b Data from Health Survey of England, 1998 Cardiovascular Disease, Table 3.13.

c Data from Health Survey of England, 1998 Cardiovascular Disease, Table 3.22 (www.official-documents.co.uk/document/doh/survey98).

.

## Appendix B1

A comparison of the baseline characteristics of study participants with EPIC-Norfolk study is given in Table B1

**Table B1:**

	**Manchester study**				**EPIC-Norfolk study**			
			Current smoker				Current smoker	
	**Never smoker**	**Former smoker**	**<15 cig day^−1^**	**⩾15 cig day^−1^**	**Never smoker**	**Former smoker**	**<15 cig day^−1^**	**⩾15 cig day^−1^**
*Men*								
No. of participants (%)	91 (39)	96 (41)	18 (8)	27 (12)	918 (34)	1463 (54)	116 (4)	20 (8)
Mean (s.d.)								
Age (years)	60.8 (2.8)	60.7 (2.8)	61.2 (2.8)	60.4 (2.5)	58.0 (8.0)	60.5 (8.4)	59.0 (8.4)	56.8 (8.0)
BMI (kg m^−2^)	26.3 (4.5)	26.6 (3.2)	25.4 (2.6)	26.3 (3.8)	26.3 (3.2)	27.0 (3.3)	26.3 (3.7)	25.7 (3.2)
Pack-years smoking^a^	—	19 (9–32)	18 (11–21)	43 (35–48)	—	11 (3–23)	18 (13–28)	30.5 (23–37)
Numbers (%)								
Major illness^b^	23 (25)	42 (44)	4 (22)	8 (30)	250 (27)	513 (35)	34 (29)	43 (21)
								
*Women*								
No. of participants (%)	110 (52)	60 (29)	19 (9)	21 (10)	1894 (56)	1127 (33)	191 (6)	173 (5)
Mean (s.d.)								
Age (years)	59.7 (2.7)	60.5 (2.7)	59.8 (2.4)	59.3 (2.7)	58.9 (8.2)	59.8 (8.7)	58.0 (8.7)	55.1 (6.7)
BMI (kg m^−2^)	26.6 (4.3)	26.9 (5.3)	25.8 (4.3)	25.8 (3.5)	26.1 (4.2)	27.0 (4.7)	25.1 (4.1)	25.3 (4.1)
Pack-years smoking^a^	—	11 (5–19)	11 (6–20)	40 (33–45)	—	5 (1–14)	13 (6–18)	25 (19–30)
Numbers (%)								
Major illness^b^	29 (26)	14 (23)	5 (26)	4 (19)	501 (27)	326 (29)	36 (19)	35 (20)
Current HRT use	42 (38)	23 (38)	8 (42)	10 (48)	291 (20)	230 (26)	38 (26)	37 (29)

s.d.=standard deviation.

a Pack-years presented as median (inter-quartile range).

b Major illness included: high blood pressure (hypertension) requiring treatment with drugs, high blood cholesterol (hyperlipidemia), angina, heart attack (myocardial infarction), stroke, other vascular disease (peripheral vascular disease), diabetes mellitus (excluding gestational diabetes) and cancer.

.

## References

[bib1] Allen NE, Appleby PN, Kaaks R, Rinaldi S, Davey GK, Key TJ (2003) Lifestyle determinants of serum insulin-like growth-factor-I (IGF-I), C-peptide and hormone binding protein levels in British women. Cancer Causes Control 14: 65–74 12708727 10.1023/a:1022518321634

[bib2] Chang S, Wu X, Yu H, Spitz MR (2002) Plasma concentrations of insulin-like growth factors among healthy adult men and postmenopausal women: associations with body composition, lifestyle, and reproductive factors. Cancer Epidemiol Biomarkers Prev 11: 758–766 12163330

[bib3] Chao A, Thun MJ, Jacobs EJ, Henley SJ, Rodriguez C, Calle EE (2000) Cigarette smoking and colorectal cancer mortality in the cancer prevention study II. J Natl Cancer Inst 92: 1888–1896 11106680 10.1093/jnci/92.23.1888

[bib4] Clemmons DR (1997) Insulin-like growth factor binding proteins and their role in controlling IGF actions. Cytokine Growth Factor Rev 8: 45–62 9174662 10.1016/s1359-6101(96)00053-6

[bib5] Department of Health (2000) Health Survey of England, 1998 Cardiovascular Disease Table 3.13 and Table 3.22

[bib6] Firth SM, Baxter RC (2002) Cellular actions of the insulin-like growth factor binding proteins. Endocr Rev 23: 824–854 12466191 10.1210/er.2001-0033

[bib7] Flexi-Scope-Trial-Collaborators (2002) Single flexible sigmoidoscopy screening to prevent colorectal cancer: baseline findings of a UK multicentre randomised trial. Lancet 359: 1291–1300 11965274 10.1016/S0140-6736(02)08268-5

[bib8] Goodman Gruen D, Barrett Connor E (1997) Epidemiology of insulin-like growth factor-I in elderly men and women. The Rancho Bernardo Study. Am J Epidemiol 145: 970–976 9169905 10.1093/oxfordjournals.aje.a009065

[bib9] Gunnell D, Oliver SE, Peters TJ, Donovan JL, Persad R, Maynard M, Gillatt D, Pearce A, Hamdy FC, Neal DE, Holly JM (2003) Are diet–prostate cancer associations mediated by the IGF axis? A cross-sectional analysis of diet, IGF-I and IGFBP-3 in healthy middle-aged men. Br J Cancer 88: 1682–1686 12771980 10.1038/sj.bjc.6600946 PMC2377147

[bib10] Holly JM, Hughes SC (1994) Measuring insulin-like growth factors: why, where and how? J Endocrinol 140: 165–169 7513340 10.1677/joe.0.1400165

[bib11] Holmes MD, Pollak MN, Hankinson SE (2002a) Lifestyle correlates of plasma insulin-like growth factor I and insulin-like growth factor binding protein 3 concentrations. Cancer Epidemiol Biomarkers Prev 11: 862–867 12223430

[bib12] Holmes MD, Pollak MN, Willett WC, Hankinson SE (2002b) Dietary correlates of plasma insulin-like growth factor I and insulin-like growth factor binding protein 3 concentrations. Cancer Epidemiol Biomarkers Prev 11: 852–861 12223429

[bib13] Jones JI, Clemmons DR (1995) Insulin-like growth factors and their binding proteins: biological actions. Endocr Rev 16: 3–34 7758431 10.1210/edrv-16-1-3

[bib14] Juul A (2003) Serum levels of insulin-like growth factor I and its binding proteins in health and disease. Growth Horm IGF Res 13: 113–170 12914749 10.1016/s1096-6374(03)00038-8

[bib15] Kaklamani VG, Linos A, Kaklamani E, Markaki I, Mantzoros C (1999) Age, sex, and smoking are predictors of circulating insulin-like growth factor 1 and insulin-like growth factor-binding protein 3. J Clin Oncol 17: 813–817 10071271 10.1200/JCO.1999.17.3.813

[bib16] Landin Wilhelmsen K, Wilhelmsen L, Lappas G, Rosen T, Lindstedt G, Lundberg PA, Bengtsson BA (1994) Serum insulin-like growth factor I in a random population sample of men and women: relation to age, sex, smoking habits, coffee consumption and physical activity, blood pressure and concentrations of plasma lipids, fibrinogen, parathyroid hormone and osteocalcin. Clin Endocrinol Oxf 41: 351–357 7955442 10.1111/j.1365-2265.1994.tb02556.x

[bib17] Lee HY, Chun KH, Liu B, Wiehle SA, Cristiano RJ, Hong WK, Cohen P, Kurie JM (2002) Insulin-like growth factor binding protein-3 inhibits the growth of non-small cell lung cancer. Cancer Res 62: 3530–3537 12068000

[bib18] Leung K-C, Johannsson G, Leong GM, Ho KKY (2004) Estrogen regulation of growth hormone action. Endocr Rev (in press) 10.1210/er.2003-0035 15466938

[bib19] Ma J, Giovannucci E, Pollak M, Chan JM, Gaziano JM, Willett W, Stampfer MJ (2001) Milk intake, circulating levels of insulin-like growth factor-I, and risk of colorectal cancer in men. J Natl Cancer Inst 93: 1330–1336 11535708 10.1093/jnci/93.17.1330

[bib20] McCaffery K, Wardle J, Nadel M, Atkin W (2002) Socioeconomic variation in participation in colorectal cancer screening. J Med Screen 9: 104–108 12370320 10.1136/jms.9.3.104

[bib21] McCaig C, Perks CM, Holly JM (2002) Intrinsic actions of IGFBP-3 and IGFBP-5 on Hs578T breast cancer epithelial cells: inhibition or accentuation of attachment and survival is dependent upon the presence of fibronectin. J Cell Sci 115: 4293–4303 12376561 10.1242/jcs.00097

[bib22] McTiernan A (2003) Intervention studies in exercise and cancer prevention. Med Sci Sports Exerc 35: 1841–1845 14600548 10.1249/01.MSS.0000093749.90499.63

[bib23] Peto R, Darby S, Deo H, Silcocks P, Whitley E, Doll R (2000) Smoking, smoking cessation, and lung cancer in the UK since 1950: combination of national statistics with two case–control studies. BMJ 321: 323–329 10926586 10.1136/bmj.321.7257.323 PMC27446

[bib24] Platz EA, Pollak MN, Rimm EB, Majeed N, Tao Y, Willett WC, Giovannucci E (1999) Racial variation in insulin-like growth factor-1 and binding protein-3 concentrations in middle-aged men. Cancer Epidemiol Biomarkers Prev 8: 1107–1110 10613344

[bib25] Pollak M (2000) Insulin-like growth factor physiology and cancer risk. Eur J Cancer 36: 1224–1228 10882860 10.1016/s0959-8049(00)00102-7

[bib26] Rajaram S, Baylink DJ, Mohan S (1997) Insulin-like growth factor-binding proteins in serum and other biological fluids: regulation and functions. Endocr Rev 18: 801–831 9408744 10.1210/edrv.18.6.0321

[bib27] Renehan AG (2004) IGFs and Cancer. www.christie.man.ac.uk/profinfo/departments/surgery/default.htm

[bib28] Renehan AG, Jones J, O'Dwyer ST, Shalet SM (2003) Determination of IGF-I, IGF-II, IGFBP-2, and IGFBP-3 levels in serum and plasma: comparisons using the Bland–Altman method. Growth Horm IGF Res 13: 341–346 14624768 10.1016/s1096-6374(03)00112-6

[bib29] Renehan AG, O'Dwyer ST, Shalet SM (2000a) RESPONSE: more about: prospective study of colorectal cancer risk in men and plasma levels of insulin-like growth factor (IGF)-I and IGF-binding protein-3. J Natl Cancer Inst 92: 1949–1950 10.1093/jnci/92.23.1949-a 11106693

[bib30] Renehan AG, Painter JE, Atkin WS, Potten CS, Shalet SM, O' Dwyer ST (2001) High-risk colorectal adenomas and serum insulin-like growth factors. Br J Surg 88: 107–113 11136321 10.1046/j.1365-2168.2001.01645.x

[bib31] Renehan AG, Painter JE, O'Halloran D, Atkin WS, Potten CS, O'Dwyer ST, Shalet SM (2000b) Circulating insulin-like growth factor II and colorectal adenomas. J Clin Endocrinol Metab 85: 3402–3408 10999841 10.1210/jcem.85.9.6770

[bib32] Renehan AG, Zwahlen M, Minder C, O'Dwyer ST, Shalet SM, Egger M (2004) Insulin-like growth factor (IGF)-I, IGF binding protein-3, and cancer risk: systematic review and meta-regression analysis. Lancet 363: 1346–1353 15110491 10.1016/S0140-6736(04)16044-3

[bib33] Sargeant LA, Khaw KT, Bingham S, Day NE, Luben RN, Oakes S, Welch A, Wareham NJ (2001) Cigarette smoking and glycaemia: the EPIC-Norfolk Study. European Prospective Investigation into Cancer. Int J Epidemiol 30: 547–554 11416081 10.1093/ije/30.3.547

[bib34] Teramukai S, Rohan T, Eguchi H, Oda T, Shinchi K, Kono S (2002) Anthropometric and behavioral correlates of insulin-like growth factor I and insulin-like growth factor binding protein 3 in middle-aged Japanese men. Am J Epidemiol 156: 344–348 12181104 10.1093/aje/kwf069

[bib35] Thissen JP, Ketelslegers JM, Underwood LE (1994) Nutritional regulation of the insulin-like growth factors. Endocr Rev 15: 80–101 8156941 10.1210/edrv-15-1-80

[bib36] Voskuil DW, de Mesquita HB, Kaaks R, van Noord PA, Rinaldi S, Riboli E, Grobbee DE, Peeters PH (2001) Determinants of circulating insulin-like growth factor (IGF)-I and IGF binding proteins 1–3 in premenopausal women: physical activity and anthropometry (Netherlands). Cancer Causes Control 12: 951–958 11808715 10.1023/a:1013708627664

[bib37] Wakai K, Ito Y, Suzuki K, Tamakoshi A, Seki N, Ando M, Ozasa K, Watanabe Y, Kondo T, Nishino Y, Ohno Y (2002) Serum insulin-like growth factors, insulin-like growth factor-binding protein-3, and risk of lung cancer death: a case–control study nested in the Japan Collaborative Cohort (JACC) Study. Jpn J Cancer Res 93: 1279–1286 12495466 10.1111/j.1349-7006.2002.tb01235.x PMC5926930

[bib38] Yu H, Rohan T (2000) Role of the insulin-like growth factor family in cancer development and progression. J Natl Cancer Inst 92: 1472–1489 10995803 10.1093/jnci/92.18.1472

